# This is a platform alteration: a trial management perspective on the operational aspects of adaptive and platform and umbrella protocols

**DOI:** 10.1186/s13063-019-3216-8

**Published:** 2019-05-29

**Authors:** Francesca Schiavone, Riya Bathia, Krishna Letchemanan, Lindsey Masters, Claire Amos, Anna Bara, Louise Brown, Clare Gilson, Cheryl Pugh, Nafisah Atako, Fleur Hudson, Mahesh Parmar, Ruth Langley, Richard S. Kaplan, Chris Parker, Gert Attard, Noel W. Clarke, Silke Gillessen, Nicholas D. James, Tim Maughan, Matthew R. Sydes, On behalf of past and present members of the STAMPEDE and FOCUS4 Trial Management Group

**Affiliations:** 1 MRC Clinical Trials Unit at UCL, Institute of Clinical Trials and Methodology, UCL, 90 High Holborn, London, WC1V 6LJ UK; 2 MRC London Hub for Trials Methodology Research, London, UK; 3 Institute of Cancer Research, Sutton, UK; 4 Royal Marsden Hospital, Sutton, UK; 5 UCL Cancer Institute, University College London, London, UK; 6 Christie and Royal Salford Hospital, Manchester, UK; 7 Division of Cancer Sciences, University of Manchester and the Christie, Manchester, UK; 8 Kantonsspital St. Gallen, St. Gallen, Switzerland; 9 Institute of Cancer and Genomic Sciences, University of Birmingham, Edgbaston, Birmingham, UK; 10 Cancer Research UK/MRC Oxford Institute for Radiation Oncology, University of Oxford, Oxford, UK

**Keywords:** Adaptive trials, Platform, Multi-arm multi stage, Protocol, Trial conduct, Trial management

## Abstract

**Background:**

There are limited research and literature on the trial management challenges encountered in running adaptive platform trials. This trial design allows both (1) the seamless addition of new research comparisons when compelling clinical and scientific research questions emerge, and (2) early stopping of accrual to individual comparisons that do not show sufficient activity without affecting other active comparisons. Adaptive platform design trials also offer many potential benefits over traditional trials, from faster time to accrual to contemporaneously recruiting multiple research comparisons, added flexibility to focus on more promising research comparisons via pre-planned interim analyses and potentially shorter time to primary results. We share here our experiences from a trial management perspective, highlighting the challenges and successes.

**Methods:**

We evaluated the operational aspects of making changes to these adaptive platform trials and identified both common and trial-specific challenges. The operational steps and challenges linked to both the addition of new research comparisons and stopping recruitment following pre-planned interim analysis were considered in our evaluation.

**Results:**

Specific operational challenges in these adaptive platform protocols, additional to those in traditional two-arm trials, were identified. Key lessons are presented describing some of the solutions and considerations over conducting these trials.

Careful consideration on the practicality of the protocol structure (modular versus single protocol), the longevity and continuity of trial oversight committees, and having clear clinical and scientific criteria for the addition of new research comparisons were identified as some of the most common challenges.

**Conclusions:**

Understanding the operational complexities associated with running adaptive platform protocols is paramount for their conduct, adaptive platform trials offer an efficient model to run randomised controlled trials and we are continuing to work to reduce further the effort required from an operational perspective.

**Trial registration:**

FOCUS4: ISRCTN Registry, ISRCTN90061546. Registered on 16 October 2013. STAMPEDE: ISRCTN Registry, ISRCTN78818544. Registered on 2 February 2004.

## Background

Adaptive trial designs are increasingly used as an efficient approach to assess research treatments [1–3]. Multi-Arm Multi-Stage (MAMS) trials are one practicable design which can contemporaneously assess multiple research treatments, often with a shared control arm, and selectively focus on more promising research comparisons via pre-planned interim analyses with in-built stopping guidelines [4, 5]. This novel design can intuitively couple with the assessment of other promising treatments in a platform protocol (sometimes known as ‘master protocol’ or ‘living protocol’), initially or later [6, 7]. Some recognised advantages compared to traditional designs include: the repeated contribution of participants, particularly in the control arm; quick initiation of sites to new comparisons; and, in many instances, reduced cost per comparisons [1, 4–6, 8].

**Fig. 1:**
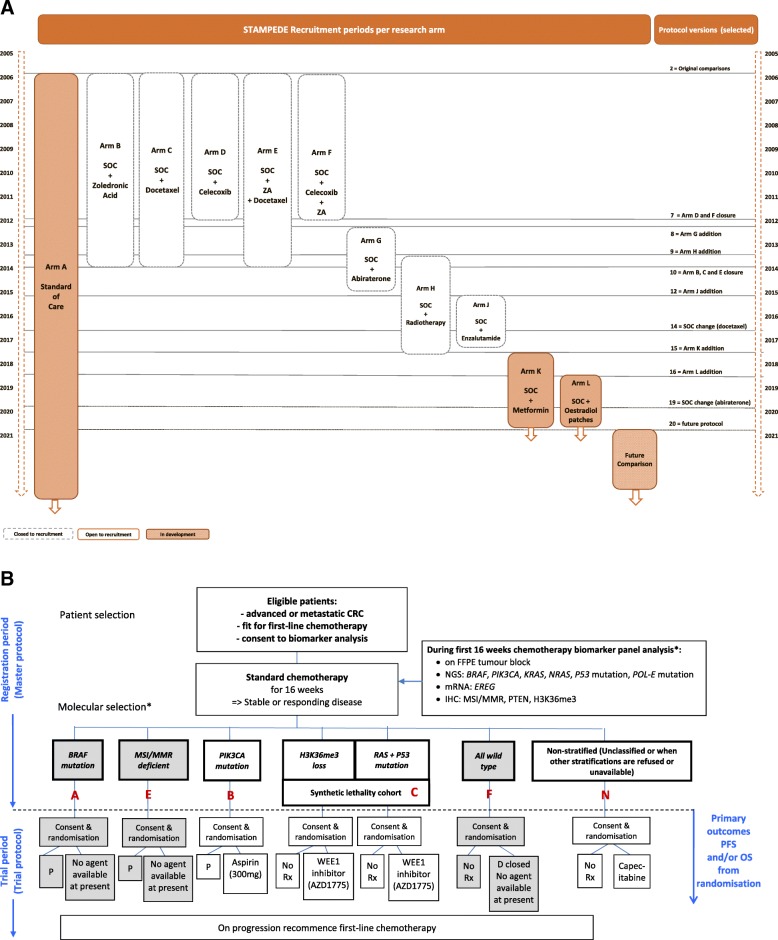
**a** STAMPEDE Trial schema. **b** FOCUS4 Trial schema

These advantages are increasingly understood by trialists and funders, but little attention has yet been given to operational and trial conduct aspects in adaptive trials. FOCUS4 (ISRCTN90061546) and STAMPEDE (ISRCTN78818544) are key examples of implementing adaptive and platform designs; both trials are sponsored by the Medical Research Council (MRC), designed and coordinated from MRC Clinical Trials Unit (CTU) at UCL; both trial designs are presented in Fig. 1a and b. FOCUS4 is a multi-site randomised trial programme using an umbrella design, including biomarker-stratified and non-stratified comparisons in one protocol. It aims to investigate novel agents, in double-blinded comparisons where possible, for patients with inoperable advanced/metastatic colorectal cancer (mCRC) [6, 9]. Since initiating in 2014 with two comparisons, FOCUS4 has opened two additional comparisons across three molecular cohorts and closed another following a pre-planned interim analysis. STAMPEDE is an international protocol investigating the efficacy of multiple treatments in advanced and metastatic prostate cancer. The trial opened in 2005 with five research comparisons and has evolved into a ‘platform’ since 2011 with the addition of six new comparisons; since trial inception, six comparisons completed recruitment and two were stopped early following interim analysis [4]. Both protocols have further comparisons in development to address emerging research questions.

Issues in stopping accrual early are already covered elsewhere [4]; here, we identify common operational components, themes and challenges in these trials so both real and perceived barriers can be overcome and adaptive designs used more widely.^1^

## Methods

We identified two well-established platform adaptive protocols at MRC CTU at UCL, STAMPEDE and FOCUS4, that have already had extensive experience of adding and dropping comparisons. A core working group from both trial management teams was formed to identify key operational components, specific to trial management and trial conduct perspectives. This working group included Trial Managers, Clinical Project Managers and Statisticians, and sought contributions from representatives of other roles, including the Unit’s Data Management System team.

Operational components were identified across the protocols, which are essential to consider when implementing a new research comparison to an ongoing protocol. These were independently identified by each member of the core working group discursively using semi-structured discussions; features for each component were discussed and 10 key components agreed upon for inclusion.

We discuss these components in the order in which they may unfold from the initial proposal to implementation of a new research comparison.

## Results

### Selection of new research comparisons

A new comparison in the STAMPEDE protocol can be considered from one perspective as starting a new trial, incorporated within an existing protocol. The scientific justification for the new research arms must be as strong as for any new trial and consideration must be given on whether it is most appropriate to do this comparison within the existing protocol rather than separately in a new protocol. Broadly, if there is substantial overlap in the patient group, reference treatment (standard of care), participating sites and primary outcome measures, embedding a new comparison can bring many efficiencies. The STAMPEDE trial team developed criteria and a selection process before STAMPEDE’s first added comparison, the ‘abiraterone comparison’, activated in November 2011. These criteria (Table 1) have evolved to guide the addition of other comparisons and, recently, criteria for biomarker-stratified comparisons. All comparisons are developed with the UK National Cancer Research Institute (NCRI) Prostate Clinical Studies Group and are independently peer-reviewed, as would be any new trial, by Cancer Research UK (CRUK), STAMPEDE’s main grant funder.

**Table 1:** MRC CTU at UCL criteria for considering addition of new research comparisons

No.	Assessment criteria
General criteria	
1.	Sound scientific rationale, including a robust biological hypothesis that supports the need to assess in the disease setting
2.	Preliminary evidence to support mechanisms of action or activity or, for combinations, synergy in the disease setting being investigated
3.	Therapy is as different as possible from other therapies in the trial (past, present and future)
4.	Clear path for the trial results to translate into improved clinical care or impact on public health
5.	Investigator enthusiasm for the new research arm and ability to recruit patient population
6.	Sufficient number of sites could be reasonably expected to meet the requirements to participate
7.	Successful independent peer review as for a new study
8.	If appropriate, relevant industry partners willing to collaborate and contribute to the trial, if the research comparison involves a pharmaceutical agent
9.	Financial sustainability: funding identified and secured to ensure central TMT support in place to support new comparison
10.	Future relevance: The results must still be relevant when the trial matures. Other trials are not already under way for this treatment or will not report sooner unless duplication, replication and/or more data are strongly required
11.	Recruitment to new comparison must not jeopardise completion of the ongoing research comparisons
12.	No implications for ongoing and future planned comparisons (e.g. overlapping populations of interest, impact on accrual)
Biomarker-specific criteria	
13.	Preliminary evidence to support the claim that a particular class of drug or specific agent has mechanisms of action or clinical activity that identifies it as a candidate within the biomarker defined subgroup. Acceptable data include: • evidence that targeting the driver pathway in the biomarker defined subgroup results in preclinical and or clinical evidence of activity; • strong epidemiological evidence of a link between an agent and a biomarker defined subgroup; • preclinical data to support a synthetic lethal interaction between a biomarker defined subgroup and an agent; • clinical data from a different disease setting for drug activity in a defined biomarker subgroup
14.	Biomarker assay characteristics: Technical ability to test for the biomarker, reproducibly with establishment of an SOP for assessment of the biomarker, with clear positive and negative controls and a quality assurance programme established between the biomarker laboratories
15.	Biomarker operating characteristics: Prevalence of biomarker likely to be sufficient to define an achievable population
16.	Prognostic and predictive effects are sufficiently well understood

FOCUS4 was planned from the start to include new comparisons. It opened in January 2014 with one randomised comparison in a molecularly defined cohort and a comparison for all patients regardless of the molecular characterisation to all eligible participants until targeted agents were confirmed for each of the other three planned molecular cohort comparisons. The molecular cohorts have evolved in response to emerging external knowledge, as have the selection criteria for biomarker-defined cohorts (Table 1). These selection criteria were agreed with FOCUS4’s funders, MRC/NIHR Efficacy and Mechanism Evaluation (EME) Programme and CRUK, and these principles have guided discussions to consider each proposed new comparison.

Assessing new comparisons requires continuous dialogue with the Trial Management Group (TMG) and potential external collaborators as well as a clear understanding of the available evidence supporting new research questions. New comparisons are not only sought with novel pharmacological treatments already tested in the specific disease type, but repurposed treatments not traditionally used in oncology setting (for example, celecoxib and aspirin or anti-diabetic drugs such as metformin) have been considered. These comparisons have been possible because of the design efficiencies of adaptive platform protocols, filling a gap in the clinical and scientific landscape in clinical research. However, intrinsic operational challenges emerge each time an additional treatment is considered. For example, there is potential for a change in the risks associated within the trial with associated changes in trial management practice. Further, the availability and accessibility of these treatments may require complex procurement, manufacturing and packaging processes.

### Clinical leadership of new comparisons

Governance structures are important for clinical trials. The TMG for each trial is responsible for the day-to-day delivery of the study and should reflect the multidisciplinary and evolving nature of platform protocols. While the Chief Investigator (CI) is appointed by the Sponsor for the overall clinical leadership to the trial platform, other appropriately qualified investigators can share these responsibilities for specific comparisons and provide the required clinical oversight specific to each comparison. Both STAMPEDE and FOCUS4 have introduced Comparison CIs (CCI) and Comparison Co-CIs (Co-CCI) for new comparisons, often someone not already on the TMG. The CCI is responsible for leading a dedicated subgroup developing the research question underpinning a new comparison, facilitating its introduction and championing it across the clinical community. Specialised advice might also be required; for example, the STAMPEDE’s TMG incorporated a diabetologist when the ‘metformin comparison’ was introduced motivated by the fact that the Investigational Medicinal Product (IMP) is not typically used in oncology settings and specialised input was warranted. In platform protocols, TMG charters require regular updating with CCIs and Co-CIs to confirm roles and responsibilities, ideally using standard templates [10]. Patient and Public Involvement (PPI) is required in the TMG to represent the patient’s voice. STAMPEDE currently has two members and FOCUS4 both a patient and a carer representative; in each trial, PPI representatives were present from the first design meetings onwards [11].

A strong, diverse and collaborative TMG is recognised as an important asset for these protocols; TMG organically expand as the science of new treatments emerge, an essential feature for the adaptive nature of platform protocols. Capacity-building can be achieved by selecting emerging researchers as CCIs.

### Scientific peer review

Each comparison in a platform protocol must be independently scientifically peer-reviewed. The core grant funder has organised this for STAMPEDE and FOCUS4, with additional review by the Trial Steering Committee (TSC), the relevant national disease research committees (specifically, the NCRI’s Clinical Studies Group in the UK) and, where relevant, industry collaborators. Proposals for new comparisons and changes to FOCUS4 require scientific peer review at a Sub-Board meeting that is represented by both funders (NIHR EME and CRUK) because they jointly funded the trial. Forward planning for the FOCUS4 EME Sub-Board meetings has been imperative, especially when there are proposals for new comparisons in the pipeline which also need to be reviewed by the TSC. Having both of these groups specific to FOCUS4 and, where possible, membership consistent throughout the life of the trial, were identified as positive strategies to ensure effective trial oversight.

Before STAMPEDE’s first added comparison (the ‘abiraterone comparison’), CRUK had no procedures and systems for peer review and funding for amendments to incorporate new comparisons. Through collaborative dialogue with the funding committee’s Chair and Research Manager, a process was developed introducing a specific amendment form for new comparisons. There is now potential for review of amendments between meetings (‘out-of-committee review’) where funding is not requested, which can greatly reduce review timelines [12].

Significant engagement early on in the trial by all key stakeholders has proven invaluable to ensure understanding the strategy, aims and design of both protocols.

### Funding

Funding for each comparison should be sought from relevant parties. By pooling them in one shared protocol, each comparison’s costs can be substantially less than they would have been as a series of traditional standalone two-arm randomised controlled trials.

CRUK provided overall grant funding for the ‘original comparisons’ in STAMPEDE and extended this for the two added comparisons (‘M1|RT comparison’ and ‘metformin comparison’) with no obvious industry partner. NIHR, MRC and CRUK have provided grant funding to FOCUS4, which was intended to support the initial platform set-up, the original biomarker panel analysis costs and operational resource for trial conduct through to completion of the trial in 2020.

Six industry partners have so far contributed to STAMPEDE (three originally, three subsequently) and two industry partners have contributed to FOCUS4. In each case, provision of investigational drugs and distribution costs were sought by the investigators.

Early dialogue with funders, ahead of submission for peer review, can ensure the necessary flexibility for all parties. Costing is a particular challenge for adaptive and platform protocols. The exact timing of the addition of new comparisons may be envisaged in principle but dates can slip for practical reasons. Therefore, added comparisons should be costed separately to ensure the protocol has adequate support throughout its lifespan.

### Biomarker development and cohort selection

Accurate molecular stratification is critical to FOCUS4; therefore, careful preparation is required. A dedicated sub-group of the TMG was formed to focus on the biomarker protocol development before the trial starting. Two academic laboratories (Leeds and Cardiff) process and analyse tumour samples for biomarkers. To ensure concordance of results and molecular cohort stratification between laboratories, a Quality Assurance (QA) process was set up for pre-trial and ongoing checks [13].

If the addition of a new research comparison also requires the assessment of new biomarkers, further funding for biomarker development may be required. Since the initiation of FOCUS4, the definition of the molecular cohorts has changed, new biomarkers have been added and there have been improvements to the analysis methodology used to detect genetic mutations. Implementation of these changes posed logistical challenges; hence, the following were considered before making the changes:

- ◦ Development time and cost for new biomarker assays in the laboratories;
- ◦ Cross-laboratory QA [13], specifically for new biomarkers and new analysis methodologies;
- ◦ Timing of implementation of changes to the biomarker panel.

Practically, changing the molecular cohort definition with existing biomarkers has an impact on the randomisation algorithm (as presented in our companion paper on the data management perspective) and has implications for the statistical analyses. A change in biomarker analysis methods also requires QA before being implemented for the trial and careful planning to ensure cost-effectiveness at the laboratories. Coordination of the biomarker panel development with regulatory application and implementation at sites can be challenging and therefore thorough preparation and constant review of timelines is required to ensure successful delivery of new comparisons.

STAMPEDE introduced biomarker screening of metastatic patients in selected centres in 2017, in anticipation of a molecularly stratified comparison in 2018. It had already demonstrated feasibility with a phenotypically defined research comparison within the overarching patient population (the ‘M1|RT comparison’).

### Protocol development

The FOCUS4 and STAMPEDE protocols have taken different approaches to protocol structure. For both trials, the protocols were based on the same template (MRC CTU at UCL protocol template, in line with the SPIRIT guidelines [14]). Using a template helps to ensure consistency in protocol structure.

The STAMPEDE protocol is structured as a single protocol and sections are amended when a new research comparison is added to include comparison-specific details (notably, to rationale, eligibility, treatment and statistics). As the trial has evolved, sections have been edited to reduce the detail on research comparisons no longer recruiting or treating patients, so the protocol primarily contains information that is current and therefore more relevant to the clinical management of study participants.

The FOCUS4 protocol uses a modular structure with a master protocol and separate comparison-specific protocols, each independently version-controlled to facilitate a straightforward update process when comparison-specific changes are made to the protocol. Maintaining consistency of information across protocol documents can be challenging. This is a particular issue for the modular approach used by FOCUS4, as often amendments must be made to specific ‘modules’ at different times.

A modular protocol structure may be more user-friendly, especially where some sites are not treating patients in all open research comparisons of the trial, as is the case in FOCUS4. This structure has sometimes been hampered key stakeholders’ understanding that FOCUS4 is a single protocol; this was a particular issue for the Ethics Committee (EC) and Competent Authority (CA).

Both approaches to protocol structure have merits and disadvantages; we therefore advise adequate discussion within the TMG to attempt to future-proof this crucial document. Explaining the rationale on the chosen protocol in the cover letter to EC and CA could also pave the way for a clearer review process by the research authorities and ultimately minimise delays that additional questions might otherwise raise.

### Ethics and regulatory assessment and version control

One key efficiency of platform approaches is the quicker initiation time achieved by adding new research comparisons through a substantial amendment compared to the traditional approach. The EC and CA in the UK have been open to reviewing and approving amendments relating to the addition of a new research comparisons for both protocols since the initial approvals.

The FOCUS4 team held discussions with EC and CA staff before the initial applications. This ameliorated some, but not all, of the challenges when the application was formally reviewed. Following a regulatory advice meeting to discuss the FOCUS4 design, leading to a substantial amendment, the CA accepted the full protocol, including the principle of future comparisons, under one Clinical Trial Authorisation (CTA); clarifying full submissions be submitted for each subsequent comparison added. Similar challenges arose with the EC, which did not initially accept the submission because of the protocol’s structure. FOCUS4 was resubmitted after discussion between the CI and EC chair and the application restructured to reiterate that FOCUS4 is one protocol.

Clear terminology is particularly important to reinforce this point; hence ‘comparison’ is used throughout protocols rather than ‘trial’ or ‘sub-trial’. This early work helped lay the foundations for new research comparisons to be submitted and approved as amendments to the existing protocol. The FOCUS4 team decided, for consistency, to use the version number and date of the master protocol section in the amendment documentation, rather than the version number of the updated, independently version-controlled module. This necessitates regular reminding to the EC, CA and sites of this approach.

Although the amendment review process has been effectively the same for both trials, some comparison-specific topics have been noted. For example, the research treatment in STAMPEDE’s ‘M1|RT comparison’ was radiotherapy rather than a drug, but still required CA approval because the protocol includes IMPs in other comparisons. Discussions with the CA regarding the documentation requirements for the amendment were key to guide the submission process for protocol amendments.

The approval timelines for a substantial amendment are shorter than those for a new application; however, the time required collating the documents to submit the amendment should not be underestimated.

### Contracts and drug supply

Like in any trial, prompt set-up of contracts with industry partners is crucial to seamless activation of added comparisons. In many trials, there is not a single template for contract negotiation, which is appropriate given the potential for bespoke needs for each comparison. Therefore, contract development can be lengthy, particularly for more novel agents and around Intellectual Property. Ensuring the legal teams understand the adaptive platform design, to which relatively few yet have exposure, can help decrease delays. We envisage this will improve as these designs become more familiar to industry.

Drug supply logistics present operational and contractual challenges when set up in parallel to active comparisons. Delays in signing agreements cause delays in IMP release for packaging and labelling and subsequent release to sites. There is a risk that the existing comparisons may finish accrual (e.g. target-attained or safety or efficacy signals), while the added comparisons are in set-up. This would require temporary suspension of recruitment to the whole platform until the new comparison is activated.

Each comparison brings a unique combination of challenges. The supplier for one FOCUS4 comparison would not meet FOCUS4’s preference of providing IMP packaged and labelled to sites, but could provide bulk IMP and matched placebo. Therefore, the CTU had to identify a separate packager and contract independently with them. The timelines may be comparable with each approach, but this approach increased the CTU’s workload. The contracting process was smoother for the next added comparison, using a previous collaborator which already had a good relationship.

STAMPEDE has several drug supply models, including separate approaches for the two abiraterone-containing arms. In its first added research comparison (‘abiraterone comparison’), abiraterone distribution was arranged via the manufacturer’s preferred vendor. The third added research comparison, the ‘enzalutamide + abiraterone comparison’, required a public tendering process needing considerable time and attention: finalising the tender; shortlisting eligible candidates; appointing the successful bidder. This process had knock-on implications and delayed the time to activation with a period > 200 days dedicated to the tendering process. This particular aspect is not uniquely required for adaptive platform protocols and could be a key activation step for the launch of any clinical trial of investigational medicinal products requiring packing and manufacturing activities. However, timelines can be significantly be affected by contract negotiation and EU tendering timelines; it is therefore essential to carefully consider implications on other research arms.

### Case report forms and database changes

Case report forms (CRFs) need to be appropriate to the comparisons. Therefore, adding a new research comparison can involve additions to, or additional, CRFs and review and update of all data management systems including and trial database. The operational steps for this are described in greater detail in our companion paper on data management in adaptive platform protocols (co-submitted).

### Site implementation

One key difference between STAMPEDE and FOCUS4 is the governance framework under which they were initiated. STAMPEDE, opened in October 2005, has seen changes in UK governance frameworks (COREC, NRES, HRA) and has not been permitted to adopt the coordinated local approval system brought in by NIHR Coordinated System for gaining NHS Permission (NIHR CSP); therefore, site activation follows an ad-hoc, trial-specific model. FOCUS4’s more recent inception meant the trial started under the NIHR CSP framework, where site acceptance for amendments may be assumed after a 35-day review period for trusts in England.

Both trials use a risk-based approach to decide which actions sites needs to complete before local implementation and opted for a site implementation model using pre-specified activation requirements (Table 2). Once all regulatory approvals are received for a substantial amendment, the central team provides sites activation pack with a timeframe to fulfil all comparison-specific activation requirements and gain local approval.

**Table 2:** Summary of key site-activation steps for new comparisons

Activation steps	Participating centres	Trial team to consider
Acceptance of substantial amendment	NHS Trust R&D to review and approve amendment; this may include: • R&D approval/Letter of no objections • signed confirmation from Principle investigator, Research Nurse, Pharmacy	• National requirements for NHS Management approval • CTU SOPs on Site Activation • Requirement for mNCA variation
Site training	Attendance to trial training may be required by: • Site Principle Investigator • Clinical Research team (research nurse, local coordinators, data managers) • Pharmacist	• Site-specific requirements based on level of participation (Patient Identification Centres, Randomising centre, follow-up only) • Development of comparison specific training material • Risk-based consideration for training documentation (self-declared, certificates) • Type of training (in person, teleconference)
Localise patient-related documents	Comparison specific PIS, CF and GP letters to be localised by each site	• Risk-adapted approach for verification of localised material (site confirmation of documents on headed paper vs emailing copies of documents on local headed paper for verification by trial team)
Update local investigator, pharmacy site file and site manuals	Sites to confirm (by signing and returning trial-specific confirmation to CTU trial team): • site file updated • updated trial manuals read and understood	• Updated indexes for files sent to sites to include comparison specific documents • trial manuals (i.e. Sample Handling and pharmacy manuals) to be updated and circulated before site activation

*CF* consent form, *RN* research nurse, *PIC* Patient Identification Centre, *PIS* Patient Information Sheet, *SOP* Standard Operating Procedure, *TMT* Trial Management Team

**Fig. 2:**
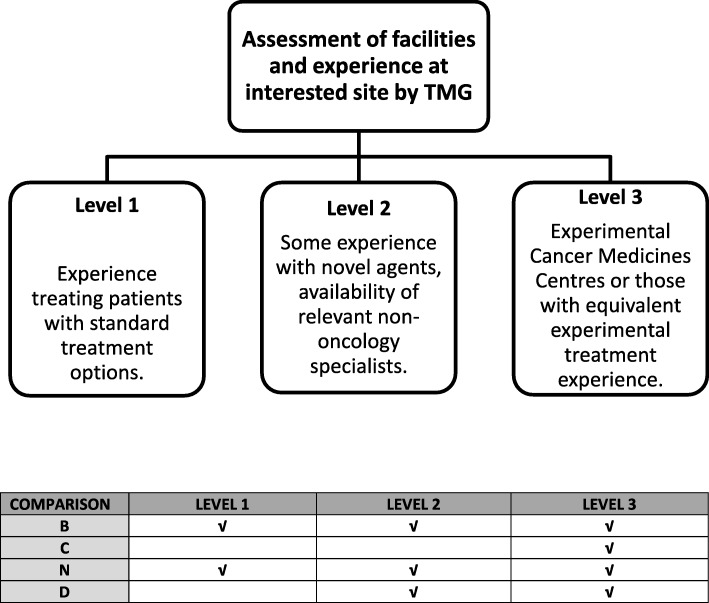
FOCUS4 site evaluation and site level classification

In FOCUS4, these requirements may also depend on the site level, as all sites identify and approach patients, even if not randomising or treating them in all comparisons (Fig. 2). Key to successful site activation was engaging with investigators sites as early as possible with email circulars and open Q&A sessions to ensure sites were aware of protocol changes and to allow time for internal feasibility.

STAMPEDE has had a shared control arm for non-biomarker-stratified comparisons, implemented in a single randomisation system. Therefore, activation had to be simultaneous across all sites. Sites not meeting all activation requirements, after several weeks’ notice, were temporarily suspended from recruitment until these were in place.

Each molecular cohort comparisons in FOCUS4 has a dedicated control arm; sites not meeting the activation requirements by the pre-set launch date were delayed from recruiting only to the new comparison and could continue registration and randomisation to other existing comparisons. The FOCUS4 team found this a labour-intensive operational process to manage, because it required manual tracking of activation status for sites.

Due to the often-novel nature of agents being considered for inclusion, the FOCUS4 selection process includes a safety assessment considering the delivery of a comparison across a widely dispersed collaborative group of sites. The FOCUS4 TMG agreed three levels of site participation, with all sites assessed before accreditation with level-specific selection criteria to account for site facilities and experience in using novel agents. Most novel agents are most appropriately administered at Experimental Cancer Medicine Centres (ECMCs) or sites assessed to have equivalent experience (FOCUS4 Level 3 site). Patients at any FOCUS4 site may be identified as suitable to consider a molecular cohort comparison using a novel agent according to their biomarker panel results and may need referral to a Level 3 site to be randomised. The likely burden for patients is considered during the selection process for new comparisons.

**Fig. 3:**
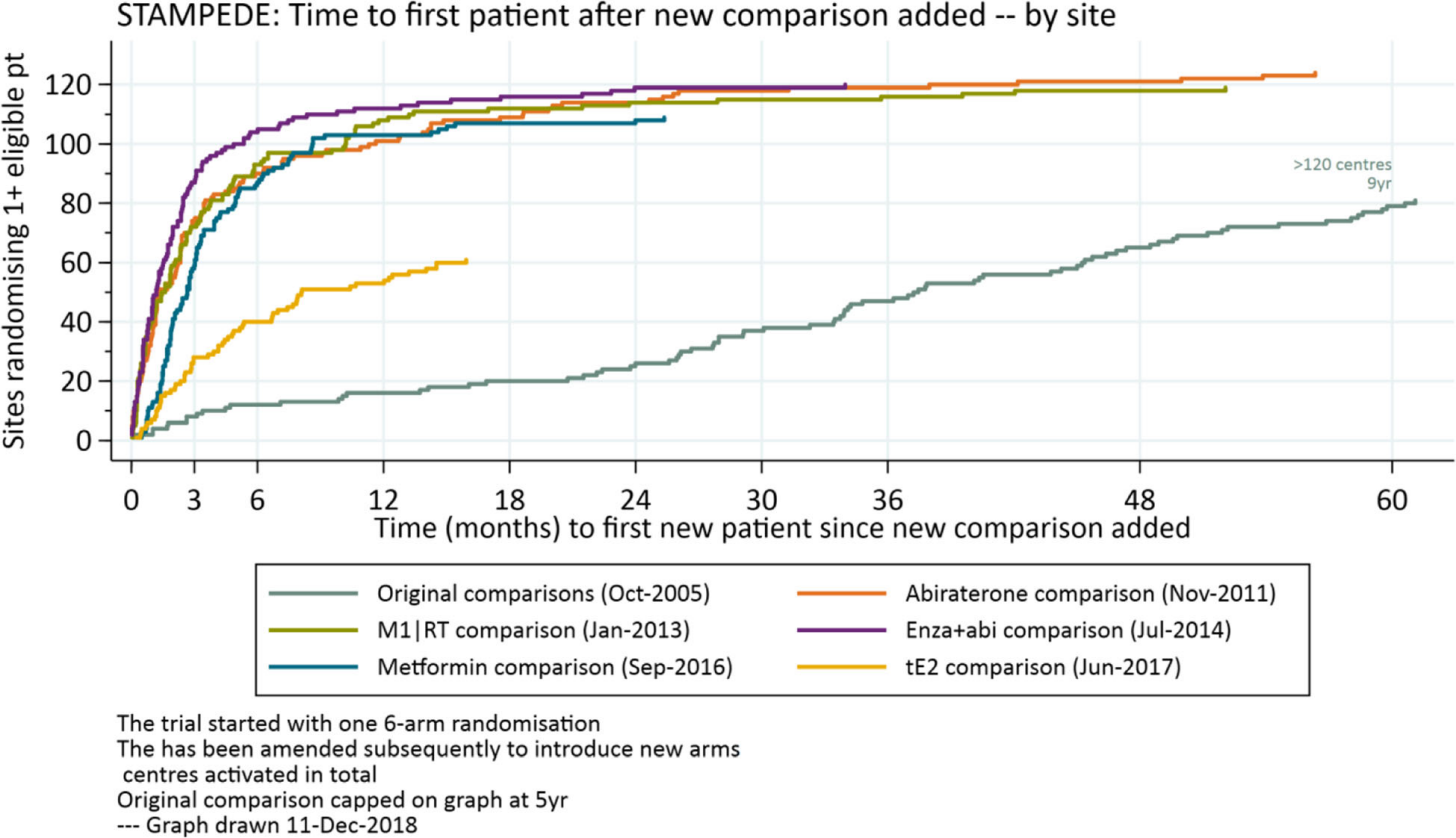
Time (months) to first new patient randomised for each STAMPEDE research comparisons. The ‘original comparisons’ (pale blue) opened slowly, deliberately activating few centres in the first 18 months, during a phase to understand acceptability of randomisation and any key toxicity signals. The ‘M1|RT comparison’ (green) only applied to a subset of patients defined by whether they have metastatic disease (~60%). The ‘tE2 comparison’ (gold), which started most recently, also applies only to a subset of patients, defined strictly by any treatment to which they have been exposed between diagnosis and randomisation

Additional comparisons were implemented in STAMPEDE without major issues. Sites reacted constructively and adapted quickly to the change showing great support; an average of 70% of centres have been ready on the day of the launch. The impact of having many sites ready to recruit to the one randomisation on activation data is demonstrated in Fig. 3 which shows the time to recruitment of comparison-eligible patients is much quicker for the additional comparisons than the original comparisons. The trial took five years to have 80 centres randomise ≥ 1 patient; the first four added comparisons had > 80 centres randomise ≥ 1 suitable patient within 3–4 months after activation. This is uncommonly quick for any trial and it is one key, practical strength of taking new comparisons into existing protocols. Each cohort has a different prevalence in FOCUS4, so this metric is less appropriate as a summary measure.

An implementation model for the addition of a new research comparison needs to balance regulatory, governance, CTU policies and SOPs as well as site capacity and capability. A one-size-fits-all approach is therefore not recommended and the TMG and TMT should think carefully about which model to adopt. An ongoing dialogue with participating centres in the months leading up to the addition of a new comparison as well as close collaboration with the trial delivery bodies (e.g. NIHR CRN in UK) has proven to be key to effectively communicating future changes and the detail of implementation plans, ultimately resulting into the success of these adaptive platform protocols.

The enthusiasm of research sites for a new comparison requires prior gauging before scientific peer review and funding has taken place to ensure a thorough initial feasibility assessment; this can be done quickly and inexpensively via online surveys, with discussion reinforced at in-person or online investigators meetings and Q&A sessions. Significant engagement early in the trial by all key stakeholders has proven invaluable to ensure understanding not only of the design but also of the strategy and aims of both protocols. It is therefore important to embed a clear communication and dissemination plan for the implementation of all new research comparisons. Short editorials published in peer-review journals are one way these trials have found to engage with the clinical and research community at large and to raise the profile and visibility of the trial platforms [15–18].

### Further considerations on trial conduct

Regular workload prioritisation exercises are advised to ensure efficient conduct; a major main trial management challenge is to balance conflicting tasks of running open comparisons while setting up new ones and, in time, closing out older ones. For example, data chasing and chasing for pre-planned interim analyses for ongoing research comparisons might coincide with activation of a new research comparison, competing for the trial team’s time or results meetings for final analyses may coincide with grant submissions for new comparisons. Indeed, the initial safety analysis for STAMPEDE’s ‘enzalutamide + abiraterone comparison’ was due six weeks after the comparison’s 100th randomisation; the high rates of accrual expected for this new comparison meant that the all CRFs and database changes had to be ready before the comparison’s activation date, whereas a separate, new, slower-recruiting trial could have afforded a more phased approach to CRF and database changes implementation. Data for this comparison needed to be actively pursued from sites to ensure timely IDMC review with patient safety being paramount.

The dynamic and adaptive nature of these trial platforms can also lead to the selective discontinuation of recruitment in a particular group cohort. For example, when recruitment to the ‘abiraterone comparison’ was completed, the only open comparison included only a subset of potential patients: only suitable for newly diagnosed metastatic patients for six months until the ‘metformin comparison’ was activated. This gap was larger than originally envisaged because of faster-than-anticipated accrual and operational challenges in activating the new comparison. Similarly, the stage I analysis for one of FOCUS4’s ‘original comparisons’ was triggered very shortly after a new research comparison was activated which pressured the trial team.

It is essential to appreciate how overall changes in platform protocols can affect the launch of a new comparison. For example, positive primary results from the STAMPEDE ‘original comparisons’ in 2016 provoked updates to the backbone standard of care [19, 20]. This required a substantial amendment to the protocol, rightly delaying activation of the ‘metformin comparison’.

Estimating the operational resources required for a platform protocol is challenging, not least because of difficulties in forecasting how long each comparison might be open. Continuity of trials unit staff is very helpful and having more experienced staff facilitates a more streamlined approach work and to workload division whereby where less training and mentoring is required.

Trial oversight is not only limited to the TMG and the commitment of members of the Independent Data Monitoring Committee (IDMC; all independent members) and the TSC (some independent members) has been a strength for these trials. The TSCs have provided impartial input to decisions on whether to add new research comparisons, with particular attention on how this would affect the integrity of existing comparisons. The longevity of the protocol means more meetings over time. FOCUS4 appointed a larger-than-usual membership, including two statisticians, allowing greater ease of assuring quoracy while anticipating scheduling difficulties for frequent meetings.

Independent oversight committees often include experienced researchers, later in their careers. Some committee members may retire in a trial that runs for many years and may need replacing. Protecting the continuity of the membership can be challenging in protocols of this magnitude, with the trial management teams nee ding to ensure all members are fully informed at all times on the protocols and any forthcoming changes.

## Discussion

Adaptive platform and umbrella protocols offer several practicable and desirable benefits which facilitate speedier answers and allow clinical trials to serve as a tool to move treatment on for patients much more quickly. There are notable operational challenges which require careful attention. Our paper reflects on some of the challenges encountered during the implementation process and crystallises our experience in the conduct of adaptive platform and umbrella protocols. Our experiences in STAMPEDE and FOCUS4 detailed in the sections above, offer some key learning points which are summarised in Table 3.

**Table 3:** Trial conduct: lessons learned on adding a new research comparison to an ongoing platform

Area	Lesson learned
Research question	Define criteria for review of new research comparison
Trial Management Group	Collaborative Group Chief Investigator: overall trial oversight Co-CI: clinical and scientific leadership for addition of new research comparison
Scientific peer review	Ongoing discussion with key funding stakeholders Planning for adequate support of central resources Addition of new comparison discussed in early stages to assess feasibility of funding
Biomarker development	Clearly define cohort and identify biomarkers Early feasibility assessment for site implementation
Protocol development	Consider protocol structure to futureproof changes in trial design (e.g. modular vs single protocol)
Ethics and regulatory approval	Rationale for addition of new comparison discussed early with regulatory bodies to prepare for submission Change of governance and regulatory framework
CRF and database development	Timelines for implementing changes are key for timely implementation
Site implementation	Engage early (e.g. via survey or Q&A) to gauge interest in new research question Discuss activation criteria with centres as early as possible Pre-set timelines for local approval of new comparison (if control arm is shared)
Other	Constant assessment of priorities and competing tasks Importance of adequate resourcing of central trial management team Consider recruitment rate to plan for post-launch activities (e.g. pre-planned interim analyses)

**Fig. 4:**
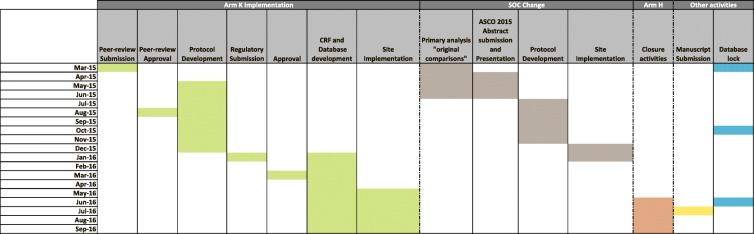
Selected STAMPEDE trial activities between March 2015 and September 2016 (research comparison addition, standard-of-care change, research comparison closure, general trial management)

A major challenge posed by running these adaptive platform and umbrella protocols is not introduced by the operational steps for the addition of a new research comparison per se, but balancing these activities against the needs of the ongoing comparisons. This was identified as a distinctive feature of this trial design, introducing a new management element over and beyond those posed by traditional two-arm trial designs. This is further presented in Fig. 4 as an example of the multiple, competing activities that required ongoing re-prioritisation and assessment.

Further work is required to improve processes and operational efficiencies to trial management so to deliver high-impact trials. Team processes need to be in place to ensure tasks are distributed appropriately and with clear communication so to ultimately successfully delivering adaptive platform protocols.

The use of shared resources across multiple comparisons must be cost-saving compared to separate two-arm non-adaptive trials to address the same questions. Efforts are required by trials units to develop costing models which can express expected costs and anticipated savings to funding bodies [8].

Recognising the capacity of sites for platform protocols is also paramount to improve trial conduct. Participating centres have dedicated enormous work to implement trials like STAMPEDE and FOCUS4 and more dialogue is needed with hospitals, ethics committees (e.g. via HRA in UK) and trial delivery bodies (e.g. NIHR CRN in UK) on the delivery of adaptive platform protocols.

More CTUs are identifying opportunities to conduct collaborative, adaptive platform protocols, including, in the UK, COMPARE in head and neck cancer (ISRCTN41478539), Precision-Panc in pancreatic cancer (http://www.precisionpanc.org/our-research/current-research/), Lung-MATRIX (NCT02664935). Each new protocol will present a unique combination of challenges. By sharing experiences, will further improve operational efficiencies.

## Abbreviations

CA: Competent authority
CCI: Co-Chief Investigator
CI: Chief Investigator
CoCCI: Co-Comparison Chief Investigator
COREC: Central Office of Research Ethics Committees
CRF: Case report form
CRUK: Cancer Research UK
CSG: Clinical Studies Group
CSP: Coordinating System for gaining NHS Permission
CTU: Clinical Trials Unit
EC: Ethics Committee
ECMC: Experimental Cancer Medicine Centre
EME: Efficacy and Mechanism Evaluation
FOCUS4: Molecular selection of therapy in colorectal cancer: a molecularly stratified randomised controlled trial programme
HRA: Health Research Authority
mNCA: Model Non-Commercial Agreement
MRC: Medical Research Council
NCRI: National Cancer Research Institute
NIHR: National Institute for Health Research
NRES: National Research Ethics Service
PPI: Patients and Participant Involvement
QA: Quality assurance
SPIRIT: Standard Protocol Items: Recommendations for Interventional Trials
STAMPEDE: Systemic Therapy in Advancing or Metastatic Prostate Cancer: Evaluation of Drug Efficacy
TMG: Trial Management Group
TSC: Trial Steering Committee
UCL: University College London

## Footnotes

1 Our lessons learnt from a central data management perspective are reported in our companion paper ‘Operational aspects of adaptive platform protocols’.
